# The COMPASS study: a longitudinal hierarchical research platform for evaluating natural experiments related to changes in school-level programs, policies and built environment resources

**DOI:** 10.1186/1471-2458-14-331

**Published:** 2014-04-08

**Authors:** Scott T Leatherdale, K Stephen Brown, Valerie Carson, Ruth A Childs, Joel A Dubin, Susan J Elliott, Guy Faulkner, David Hammond, Steve Manske, Catherine M Sabiston, Rachel E Laxer, Chad Bredin, Audra Thompson-Haile

**Affiliations:** 1 School of Public Health and Health Systems, University of Waterloo, 200 University, Avenue, Waterloo, ON N2L 3G1, Canada; 2 Department of Statistics and Actuarial Science, University of Waterloo, Waterloo, Canada; 3 Faculty of Physical Education and Recreation, University of Alberta, Waterloo, Canada; 4 Ontario Institute for Studies in Education, University of Toronto, Toronto, Canada; 5 Faculty of Kinesiology and Physical Education, University of Toronto, Toronto, Canada; 6 Propel Centre for Population Health Impact, University of Waterloo, Waterloo, Canada

**Keywords:** Obesity, Physical activity, Tobacco, Substance use, Diet, Policy, Longitudinal, Built environment

## Abstract

**Background:**

Few researchers have the data required to adequately understand how the school environment impacts youth health behaviour development over time.

**Methods/Design:**

COMPASS is a prospective cohort study designed to annually collect hierarchical longitudinal data from a sample of 90 secondary schools and the 50,000+ grade 9 to 12 students attending those schools. COMPASS uses a rigorous quasi-experimental design to evaluate how changes in school programs, policies, and/or built environment (BE) characteristics are related to changes in multiple youth health behaviours and outcomes over time. These data will allow for the quasi-experimental evaluation of natural experiments that will occur within schools over the course of COMPASS, providing a means for generating “practice based evidence” in school-based prevention programming.

**Discussion:**

COMPASS is the first study with the infrastructure to robustly evaluate the impact that changes in multiple school-level programs, policies, and BE characteristics within or surrounding a school might have on multiple youth health behaviours or outcomes over time. COMPASS will provide valuable new insight for planning, tailoring and targeting of school-based prevention initiatives where they are most likely to have impact.

## Background

Substance use and health behaviours linked to obesity (i.e., diet and physical activity) tend to be established during adolescence with most Canadian youth exhibiting one or more modifiable risk factors for future chronic disease [1]. Moreover, research suggests that within the Canadian context, characteristics of the school are independently associated with these negative behavioural outcomes among youth. For instance, significant between-school variability has been identified among Canadian youth for obesity [2], physical activity [3], sedentary behaviour [4], alcohol and marijuana use [5], and smoking [6]. Although the school context appears to be an important determinant of behaviour [7], comprehensive inventories of school characteristics that may impact behavioural development (i.e., programs, policies, and built environment resources within or surrounding schools) are typically not systematically collected or examined in school-based prevention research [8]. Few researchers have the data required to adequately understand how the school environment impacts youth health behaviour development. This has resulted in a knowledge gap in best-practice interventions for youth populations despite ongoing calls for integrated ‘whole school’ prevention strategies [9-14].

Local data collection and feedback systems have been shown to be essential when building capacity to integrate research, evaluation, policy, and practice within school-based prevention systems [8,15]. In response, we developed the COMPASS study (http://www.compass.uwaterloo.ca). The objective of COMPASS is to develop and implement a comprehensive research, evaluation and knowledge exchange system that incorporates a whole-school approach to school-based prevention programming. COMPASS uses a rigorous longitudinal quasi-experimental design to evaluate how changes in school programs, policies, and/or built environment (BE) characteristics are related to changes in multiple youth health behaviours and outcomes. COMPASS also facilitates knowledge transfer and exchange by annually providing each participating school with customized knowledge exchange tools and access to a knowledge broker to help connect them to relevant prevention resources. This paper outlines the design features, data collection methods and tools, knowledge exchange procedures, and analytical strategies of the COMPASS study.

## Methods/Design

COMPASS is a prospective cohort study designed to collect hierarchical longitudinal data from a convenience sample of secondary schools and the grade 9 to 12 students attending those schools. In Year 1 (2012–13), data were collected from 43 Ontario schools and over 24,000 grade 9 to 12 students. The Year 1 school-level sample size was lower than our original target of 90 schools due to teacher job action in Ontario Public schools during school recruitment^a^. The impact on our design and power was mitigated by recruiting new schools into the cohort in Year 2 (these new schools will still have 3 waves of data by Year 4). As such, in Year 2 (2013–14), the cohort was increased by 47 additional schools to reach our target of 90 schools (79 in Ontario and 11 in Alberta), with more than 50,000 grade 9 to 12 students participating. Our reason for recruiting schools in Alberta in Year 2 was to add value to the study design by allowing for the quasi-experimental evaluation of provincial policies and/or programs between Ontario and Alberta schools over time. The school-level sample size of 90 schools meets power requirements suggested by experts on multi-level studies [16,17] and exceeds those of publications employing multi-level models [2-6].

Given the hierarchical longitudinal nature of the data, the cohort of 90 secondary schools are being followed over time through annual school data collection of the program and policy environment within each school, the built environment characteristics within each school, and the built environment characteristics in the community immediately surrounding each school. At the student-level, the cohort of grade 9 to 12 students within the 90 schools are followed over time using annual surveys that assess obesity, healthy eating, physical activity, sedentary behaviour, tobacco use, alcohol and marijuana use, school connectedness, bullying, and academic achievement using scientifically supported measures.

We purposefully selected a longitudinal quasi-experimental design of this research given that it provides robust internal control (at the student- and school-level as a function of the longitudinal design) when examining change over time [18], and robust external validity (as a function of the quasi-experimental design). For instance, considering that much of the available school-based prevention evidence is derived from artificially controlled research which does not align with the realities of prevention practice within the school environment [19-23], COMPASS can evaluate the ‘real-world’ effectiveness of evidence-based interventions that are implemented in COMPASS schools throughout the course of the study. Considering that schools also often implement innovative and unique programs or policies that are not yet evidence-based, COMPASS can start to generate practice-based evidence by evaluating those natural experiments throughout the course of the study.

### School board and school recruitment

Since the COMPASS study is not designed to represent a geographical population outside the selected schools, Ontario and Alberta school boards and schools were purposefully sampled. Eligible schools were approached after a board granted approval. Board-level inclusion criteria required being an English-speaking secondary school board that permits the use of active-information passive-consent parental permission protocols. Inclusion criteria at the school-level included being a secondary school with students in grades 9 to 12 with a student population of at least 100 students or greater per grade that permits the use of active-information passive-consent parental permission protocols. The appropriateness of passive-consent protocols for youth surveys measuring self-reported risk behaviour has been documented previously [24-27]. Boards and schools declined to participate primarily due to competing research demands. The University of Waterloo Office of Research Ethics and appropriate School Board committees approved all procedures, including passive consent.

### Student-level recruitment

In participating schools, eligible students were recruited using active-information passive-consent permission protocols. In this approach, the parent(s) or guardian(s) of an eligible student were mailed an information letter about the COMPASS study and were asked to either (a) call the COMPASS recruitment coordinator using the 1–800 phone number provided in the information letter, or (b) email the COMPASS recruitment coordinator using the COMPASS email address provided in the information letter should they not want their child to participate. All eligible students whose parent(s) or guardian(s) did not withdraw their child were deemed eligible to participate. At any time during the consent process or during the data collection, an eligible student was allowed to decline to participate or withdraw from the study.

### Data collection tools

Given the hierarchical nature of the data, the COMPASS Student Questionnaire (C_q_) was used to collect the student-level data, and the COMPASS School Programs and Policies Questionnaire (SPP), the COMPASS School Environment Application (Co-SEA), and the COMPASS Built Environment Data (C-BED) were used to collect the school-level program, policy, and built environment data.

### COMPASS student questionnaire (C_q_)

The student-level questionnaire for COMPASS (C_q_) was designed to facilitate multiple large-scale school-based data collections consistent with previous research [2,6,28]. The C_q_ collects individual student data pertaining to obesity (height and weight to calculate body mass index [BMI]), sedentary behaviours, physical activity and evidence-based correlates of physical activity, healthy eating and diet, tobacco use, alcohol and marijuana use, bullying, academic outcomes, amount of sleep, and demographic characteristics (e.g., age, gender, income, ethnicity). Survey items were specifically chosen to reflect both science-based (e.g., obesity) and practice-based (e.g., bullying) concerns. Because the C_q_ collects data from large whole-school samples during class time, it was purposefully made short (12-pages allows it to be completed in one 30–40 minute class period), and inexpensive (machine-readable forms). The C_q_ items have demonstrated reliability and validity [29-31], and are consistent with measures used in national surveillance tools or to measure current national public health guidelines [28,32-34]. Consistent with other research [35], the cover page of the C_q_ contains measures required to create a unique self-generated code for each respondent in a school to ensure the anonymity of the survey participants, while still allowing COMPASS researchers to link each student’s unique anonymous identifier data over multiple years. Eligible students completed the C_q_ in class on the day of the scheduled data collection for their school at a time coordinated by COMPASS staff and school administration.

### COMPASS school programs and policies questionnaire (SPP)

The COMPASS School Programs and Policies Questionnaire (SPP) is a paper-based survey completed by the school administrator(s) most knowledgeable about the school program and policy environment. The SPP was based on the previously validated Healthy School Planner tool [36], but modified to be shorter in length and to cover additional content domains. The SPP measures the presence or absence of relevant programs and/or policies, and changes to school policies, practices, or resources that relate to student health for each of the behavioural domains measured in the C_q_. The completed SPP from each school was collected by COMPASS staff at the time of their school’s student-level data collection along with copies of the relevant policy handbook(s) or rules for additional document review if required.

### COMPASS school environment application (Co-SEA)

The COMPASS School Environment Application (Co-SEA) [37] is a direct observation tool that was developed to measure aspects of the built environment within a school that are associated with obesity, eating behaviour, and physical activity (the student behaviours most influenced by the built environment within a school) [38,39]. As described elsewhere [37], Co-SEA is a downloadable software application that contains an automated computer-based version of the previously validated audit measures from the ENDORSE study [39] (measuring the school food environment) and the SPEEDY study [38] (measuring the school physical activity environment), with the additional functionality of taking pictures during the audit and linking them to the data file as objective observations.

### COMPASS built environment data (C-BED)

Data on the community built environment surrounding each school (e.g., distribution of recreation centres, tobacco retailers, fast food restaurants) are provided annually by the CanMap RouteLogistics (CMRL) spatial information database and the Enhanced Points of Interest (EPOI) data resource from the Desktop Mapping Technologies Inc. (DMTI) [40]. The CMRL provides various data layers surrounding each school (e.g. land use, boundary files, and street networks) and the EPOI provides data on the type and location of different opportunity structures surrounding each school (e.g. grocery stores, fast-food restaurants, and fitness centres). Consistent with previous research [41], the process of identifying and linking the DMTI built environment data to 90 COMPASS schools involves geocoding the address for each COMPASS school, creating 500 m and 1-km circular buffers (i.e., bounded areas surrounding each school in which the different built environment characteristics were quantified), and linking the quantified built environment data for each school to those buffers using Arcview 3.3 software [42].

## Discussion

Health-promoting schools are empowered to take responsibility for promoting the health of their student population according to their needs and priorities, rather than always having to be reactive to outside regulatory bodies [43]. As such, research has identified that providing schools with data on their student population and recommendations for action that are relevant to their school context can help schools advance their own prevention agenda [9,15]. In order to help foster health promoting schools to develop stronger links and engagement with participating schools, and track knowledge use as it unfolds from inception through decision-making, adoption, adaption and implementation in participating schools, the COMPASS study developed the COMPASS School Health Profile (SHP) and connects participating schools with a COMPASS knowledge broker.

### COMPASS school health profile (SHP)

The provision of the customized school-specific COMPASS School Health Profile (SHP) to participating schools is an important knowledge exchange component of COMPASS. Each school-specific SHP report provides data on the various student health behaviours or outcomes of interest, makes comparisons to provincial and/or national norms or guidelines, and offers: (a) evidence-based suggestions for interventions, programs, or policies aimed at improving the health of the student population; (b) suggestions for curriculum supplements aimed at improving student awareness and knowledge to encourage and enable them to make healthier lifestyle choices; and (c) local Public Health Unit (PHU) (i.e., the local/regional board of health) contact information specific to each content domain, in case the school wishes to contact their local public health unit for support in taking action on the findings. The SHP allows school stakeholders to quickly see, “at a glance,” what is happening at their school and where to target future school programs and intervention activities and efforts. The SHP is generated using an automated, quality-controlled system that accurately scans data from the C_q_, inputs the data into analysis programs, and exports these data into a format that can be inputted into an existing feedback report template. This ensures that schools have up-to-date school-specific evidence pertaining to the student health behaviour(s) or outcomes of interest for their student population.

### COMPASS knowledge broker

Community of Practice principles [44] indicate that knowledge producers and users must jointly determine “better practices” for their settings. The utility of the SHP is enhanced by the availability of COMPASS knowledge brokers who have experience in both research and public health or education to: (a) facilitate interaction between our research team, school stakeholders, and community partners (e.g., the PHU responsible for each participating school in COMPASS has been engaged as a partner); (b) assist school stakeholders in determining appropriate evidence-informed action based on the SHP; and, (c) collect process measures from school stakeholders pertaining to interventions that were implemented as a function of the SHP. The knowledge brokers use a reflective practice approach, facilitated by electronic meetings (teleconferences/Skype) to build capacity for evidence-informed practice in participating schools and to collect process measures to assist with the evaluation of the impact of any implemented interventions. The reflective practice approach is consistent with “improving” population health practice as opposed to just focusing on proving effectiveness. By using a knowledge broker to develop stronger links and engagement with participating schools, we will also be able to track knowledge use from program inception through decision-making, adoption, adaption and implementation in participating schools. The goal is to engage stakeholders in reflective practice to contribute to furthering prevention science and their own evidence-based practice (i.e., knowledge exchange).

### Implications for school health

#### Analytical strategies

The hierarchical longitudinal nature of the COMPASS data allows for a number of different analytical strategies for examining each of the outcomes in COMPASS. For instance, both cross-sectional and longitudinal core analytical approaches to examining the data will be used.

Cross-sectional analyses include, but are not limited to:

1. Identification of high-risk individuals or high-risk school environments;

2. Examination of between-school variability in the different student-level outcomes among students;

3. Examination of the co-occurrence of different outcomes; and,

4. Hierarchical analyses examining the student- and school-level characteristics associated with each outcome.

Longitudinal analyses include, but are not limited to:

1. Examination of the temporal sequence for the development of individual outcomes or the co-occurrence of outcomes;

2. Hierarchical examination of how changes in school-level characteristics (programs, policies, or built environment resources) are related to changes in school-level prevalence or individual student-level outcomes over time;

3. Evaluation of how the different knowledge exchange strategies impact the provision of school-level prevention activities or resources; and,

4. Examining how the trajectories of different outcomes are predicted by other outcomes (e.g. declines in physical activity over time impact obesity) and the available sociodemographic characteristics of students and/or schools.

As described previously [45], the benefit to this type of hierarchical multi-year data is that the data can be used as either a series of cross-sectional samples at the student- and school-level or as a longitudinal sample with repeated measures at both the student- and school-levels. These data also allow for the quasi-experimental evaluation of natural experiments that will occur within schools over the course of COMPASS, providing a means for generating “practice based evidence” in school-based prevention programming [19]. Although this evidence may be considered imperfect to some because it is not a randomized control trial, it is often considered more relevant to school stakeholders since it identifies ‘effective’ interventions in real-world settings and reflects the realities of intervention implementation [8,9,19]. The design also permits more in-depth qualitative and quantitative studies to better understand school-level results.

### Opportunities for collaboration

In order to ensure that the COMPASS resources and infrastructure have impact and provide the largest scientific contribution possible, we are actively recruiting additional stakeholders, trainees, and investigators from across Canada and internationally to become engaged in COMPASS by sharing the data. To foster such engagement, we have a COMPASS Data Usage Application on the COMPASS website as a means for individuals or groups to request access to the COMPASS data.

### Attrition plan

As COMPASS unfolds beyond baseline, two approaches for dealing with attrition have been planned. First, at the student-level, the cohort of grade 12 students will graduate at the end of each school-year making them unavailable for subsequent follow up. To address this issue and other forms of student attrition (i.e., students changing schools, a student missing a data collection, etc.), each student in participating schools will be re-recruited annually into the cohort via the passive parental consent procedures. Not only does this allow for recruitment of new students to the school or those who missed earlier data collection, this approach also allows recruitment of the new cohort of grade 9 students entering a school during each subsequent wave of data collection. This approach allows for the maintenance of the student-level sample size while also maintaining ethical responsibilities of the researchers. The second plan is for dealing with potential attrition at the school-level. To prevent schools from leaving the cohort, we work very closely with participating schools and stakeholders to maintain ongoing partnerships and to ensure the study continually adds value to participating schools.

In conclusion, the COMPASS study is among the first of its kind internationally to create the infrastructure to robustly evaluate the impact that changes in school-level programs, policies, and built environment resources might have on multiple youth health behaviours and outcomes over time. Determining the school-level characteristics that are related to the development of multiple modifiable youth health behaviours and outcomes will provide valuable insight for informing the future development, tailoring, and targeting of school-based prevention initiatives to where they are most likely to have an impact [46], and will provide the opportunity to understand how the school environment can either promote or inhibit health inequities among subpopulations of at-risk youth. Such insight could save valuable and limited prevention/promotion resources. Developing the ability to evaluate natural experiments that occur within schools will substantially add to the breadth of our understanding of what interventions work, for which students, and in which context.

## Endnote

^a^The lower school-level participation rate in 2012–13 was due to teacher labour issues in Ontario public school boards as a result of the Ontario government’s Bill 115 at the time of the board and school recruitment (http://www.ontla.on.ca/web/bills/bills_detail.do?BillID=2665).

## Abbreviations

Passive consent: Active-information passive-consent; CIHR: Canadian Institute for Health Research; Cq: COMPASS Student Questionnaire; SPP: COMPASS School Programs and Policies Questionnaire; Co-SEA: COMPASS School Environment Application; C-BED: COMPASS Built Environment Data; BMI: Body Mass Index; DMTI: Desktop Mapping Technologies Inc; CMRL: CanMap RouteLogistics; EPOI: Enhanced Points of Interest; SHP: COMPASS School Health Profile.

## Competing interests

All authors declare that they have no competing interests.

## Authors’ contributions

STL conceived of the COMPASS study and wrote the funding proposal, developed the study tools, is leading the study implementation and coordination, and drafted this manuscript. KSB participated in the analytical design of the study and helped to draft the analytical strategies for this manuscript. VC helped expand the study to Alberta, is leading the study coordination in Alberta, and helped to draft this manuscript. RAC helped to draft the education outcome strategies of the study, the Cq and SPP, and helped to draft this manuscript. JAD is contributing to the analytical components of the study and helped to draft the analytical strategies for this manuscript. SJE participated in the design of the C-BED data, the SHP, and helped to draft this manuscript. GF participated in the design of the study, the Cq and SHP, and helped to draft this manuscript. DH participated in the design of the SPP, the Cq, and helped to draft this manuscript. SM participated in the design of the SPP and helped to draft this manuscript. CMS is contributing to the analytical components of the study and helped to draft this manuscript. REL participated in the design of the knowledge broker component of the study, is one of the study knowledge brokers, and helped to draft this manuscript. CB is the study manager and helped to draft the methods of this manuscript. ATH is one of the study knowledge brokers, is coordinating the study expansion in Alberta, and helped to draft the methods of this manuscript. All authors read and approved the final manuscript.

## Pre-publication history

The pre-publication history for this paper can be accessed here:

http://www.biomedcentral.com/1471-2458/14/331/prepub
